# Contrast Agent Microbubble Jetting during Initial Interaction with 200-kHz Focused Ultrasound

**DOI:** 10.1016/j.ultrasmedbio.2019.08.005

**Published:** 2019-11

**Authors:** Sarah Cleve, Claude Inserra, Paul Prentice

**Affiliations:** ⁎ Université Lyon, École Centrale de Lyon, INSA de Lyon, CNRS, LMFA UMR 5509, Écully, France; † Université Lyon 1, Centre Léon Bérard, INSERM, LabTAU, Lyon, France; ‡ CavLab, Centre for Medical and Industrial Ultrasonics, University of Glasgow, Glasgow, United Kingdom

**Keywords:** Microbubble, Jetting, Focused ultrasound, Cavitation

## Abstract

The initial response of microbubbles flowing through a 500-μm polycarbonate capillary to a burst of 200-kHz focused ultrasound, at peak-negative pressure amplitudes from 0.7–1.5 MPa, was investigated with dual-perspective high-speed imaging. Directed jetting through the acoustic focus is demonstrated according to the pressure gradients acting across the cavitating microbubbles. At lower amplitudes, repeated microbubble-jetting is accompanied by sudden, intermittent translation. At higher amplitudes a rebound jet also forms, before disintegration into a cavitation cloud.

## Introduction

Contrast agent microbubble response to focused ultrasound at sub-megahertz frequencies is of significant interest for the emerging application of cavitation-mediated opening of the blood–brain barrier (Lipsman et al. 2018). Driving frequencies (*f*_0_ values) of several hundred kilohertz, an order of magnitude lower than typical microbubble resonance, are necessary for sufficient transmission across the skull for transcranial therapy. Although a significant body of literature exists describing microbubble response to insonation parameters more typical of those associated with diagnostic imaging, at frequencies around microbubble resonance and above (*e.g.*, Chomas et al. 2002), *via* high-speed optical imaging, microbubble cavitation under such subresonant driving is less well studied. One recent report (Ilovitsh et al. 2018) investigated microbubble response to a short burst of 250-kHz focused ultrasound at peak-negative pressure (PNP) amplitudes in the range of several 100 kPa. Streak imaging indicated expansion ratios (maximum radius *R*_ma_*_x_*: equilibrium radius *R*_0_) >30, for in-house-prepared microbubbles, with *R*_0_ values between 0.75 and 1.5 μm. Streak capture, however, does not fully reveal the evolution of the driven bubble morphology in the two spatial dimensions associated with conventional optical imaging.

The host laboratory for this work has recently described a dual-high-speed imaging configuration (Song et al. 2019) for observing microbubble cavitation from dilute samples of SonoVue contrast agent in response to a 200-cycle burst of propagating focused ultrasound, at *f*_0_ = 692 kHz. Imaging from one high-speed camera was used to observe microbubble cavitation over the duration of the focused ultrasound exposure. A second high-speed camera was used to probe cavitation activity for limited durations within the burst, at high temporal resolution. In this Technical Note, we report on the use of the same experimental configuration to study the initial interaction between focused ultrasound from the same transducer, but here, operating at its lowest resonance frequency of *f*_0_ = 200 kHz, and contrast agent microbubbles. We also illustrate the effect of the initial microbubble position, through the acoustic focus, on the interaction.

## Methods

**Fig. 1:**
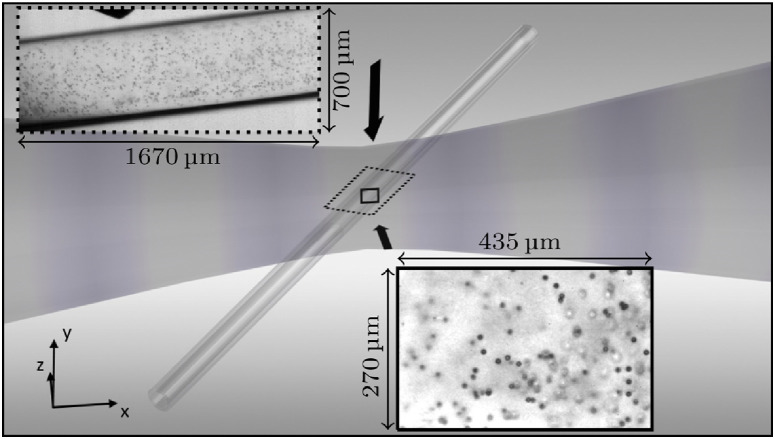
Schematic of the exposure configuration, with the capillary orientated at 45° to the propagation axis, with propagation from left to right, for dual-perspective high-speed imaging (fields of view are represented by *dotted/solid rectangles*). Insets are images of 7.5 ± 0.2-μm polymer beads (Bangs Laboratories, USA), from top view and side view perspectives, for scale and resolution.

The experimental arrangement is fully described in Song et al. (2019) and is represented schematically in Figure 1. Briefly, a polycarbonate capillary 500 μm in internal diameter and 25 μm wall thickness (Paradigm Optics, Vancouver, WA, USA) was positioned at 45° to the propagation axis of a focused ultrasound transducer, horizontally across the focal region. The transducer (H-149, Sonic Concepts, Bothell, WA USA), mounted on an xyz-manipulator, geometrically focuses to 68 mm from the front face, in the *x*-direction (Fig. 1), within a custom-made tank measuring 420  ×  438  ×  220 mm^3^ filled with degassed, de-ionised water. Phials of SonoVue (Bracco, Milan Italy) contrast agent were reconstituted daily, according to manufacturer specifications, with samples diluted by a factor of ∼ 1:80,000 in de-ioinsed water, prepared on an hourly basis. A syringe pump flowed samples through the capillary at the rate of 11 mL/h.

A high-speed camera (Fastcam SA-Z 2100 K, Photron, Bucks UK), mounted above the capillary (top view [T-V] perspective), records the interaction between a 10-cycle burst of 200-kHz focused ultrasound, and any microbubbles in the capillary at the time of incidence, at 210,000 frames per second (fps) and a shutter time of 159 ns. Imaging was undertaken through a 5  ×  long-working-distance lens (0.14 NA, Mitutoyo, Kawasaki Japan), with illumination provided by a 150-W halogen bulb coupled to a liquid light guide. The role of T-V imaging, with field-of-view (FOV) represented by the *dotted rectangle* in the horizontal xz plane (Fig. 1), and spatial resolution of ∼4.1 μm/pixel, is primarily to determine the number and relative proximity of microbubble cavitation events within the ∼1.5-mm observable length of the capillary, once initiated on focused ultrasound incidence. A second high-speed camera (Shimadzu HPV-X2, Kyoto Japan) imaging at 10 million fps over a duration of 25.6 μs, from a side-view (S-V) perspective, captures microbubble cavitation response over the first 5 cycles of driving, at high temporal resolution. Illumination was achieved with synchronous (to frame capture) 10-ns laser pulses, coupled to a liquid light guide and a collimator lens. Other than the focused ultrasound driving *f*_0_ of 200 kHz used here, the only other difference to the configuration previously described (Song et al. 2019) is that side-view imaging for this work was conducted through a 20  ×  long-working-distance objective lens (0.42 NA, Mitutoyo), at a higher spatial resolution of ∼ 1.1 μm/pixel but over a reduced FOV, represented by the *solid rectangle* in the xy plane (Fig. 1).

The PNP amplitudes quoted in the Results are the highest values measured within the field generated for any given electronic settings, through manual scanning of a 0.2-mm polyvinyl difluoride needle hydrophone (Precisions Acoustics, Dorchester UK), also mounted on an xyz-manipulator. This serves to identify the focal spot for subsequent capillary alignment, *via* T-V imaging. Further measurements, with the hydrophone tip located ∼1.5 mm into the far field, with and without the capillary in place, indicated cross-capillary attenuation of <5%.

**Fig. 2:**
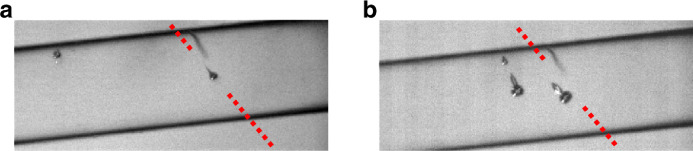
Top view images of initial microbubble cavitation response, at *t* ≈ 11.4 μs, to a focused ultrasound burst of peak negative pressure = 1.5 MPa, with the propagation axis depicted by the red dashed line, for (a) a relatively isolated microbubble located centrally within the capillary, and (b) three microbubbles, two located centrally within the capillary and one closer to the wall. Flow within the capillary is left to right. Scale is provided by the internal diameter of 500 μm, and a flaw on the capillary (*white arrowhead*) acts as a convenient fiducial.

**Fig. 3:**
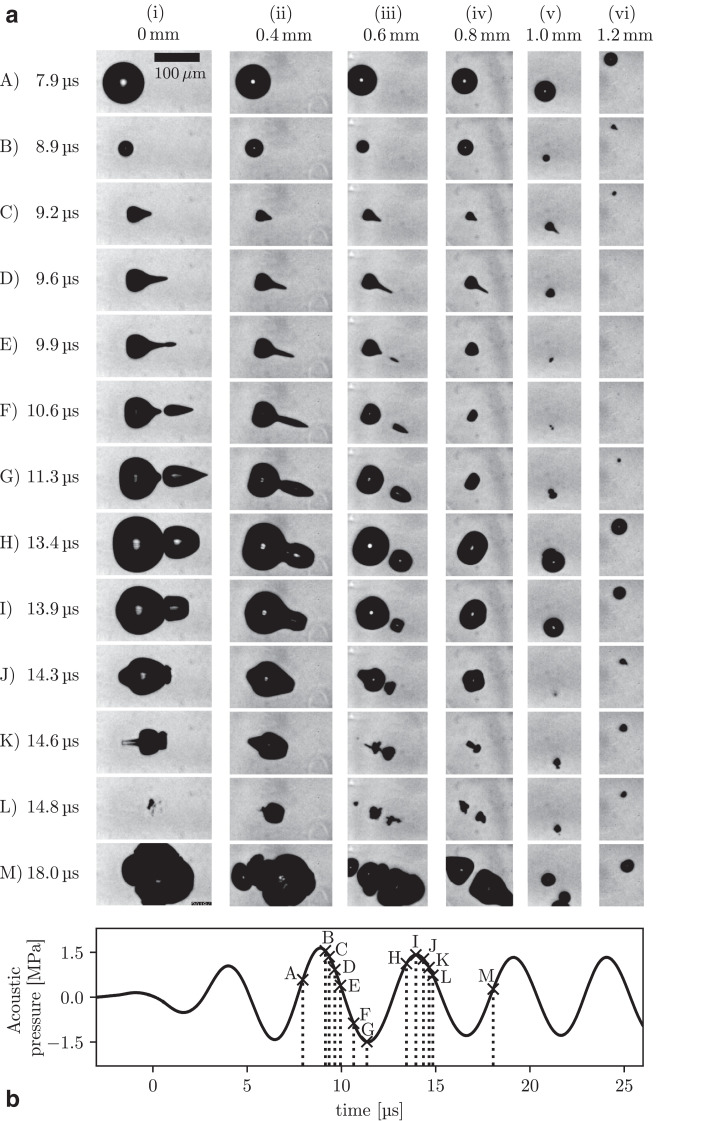
(a-i–vi) Representative images from side view imaging sequences, capturing microbubble cavitation activity from the first significant inflation, over the two subsequent cycles of focused ultrasound, at various lateral offset distances from the propagation axis. (b) An on-axis needle hydrophone measurement of the first cycles of the focused ultrasound burst (over the duration of side view imaging), at a peak negative pressure of 1.5 MPa.

The experiment was repeated until data sets were collected whereby the initial interaction between the focused ultrasound burst and a relatively isolated microbubble (as confirmed *via* T-V imaging, see Fig. 2) was imaged within the comparatively small FOV of the S-V perspective. For the directed-jetting observations of Figure 3a, the vertical y positioning of the transducer was adjusted in ±0.1-mm increments, to investigate the dependence of the interaction with initial microbubble position, laterally through the focus of the ultrasound field, with at least three data sets collected for each transducer/focus position. *t* = 0 μs is defined as the start of S-V capture, with transducer excitation and T-V imaging electronically triggered at *t* ≈ –60 μs.

## Results

Figure 2 illustrates the requirement to observe initial cavitation response from a relatively isolated microbubble, confirmed *via* T-V imaging. Figure 2a, with full image sequence available as Supplementary Video S1 (online only), captures the response of a microbubble located close to the axis of the focused ultrasound (with the transducer at y-position = 0 mm). A second microbubble is apparent within the FOV, ∼1 mm upstream (to the capillary flow) to the on-axis microbubble and close to the capillary wall. Microbubble jetting along the axis of focused ultrasound propagation, described in detail below, is taken to indicate the on-axis microbubble is sufficiently isolated such that its response is unaffected by the second microbubble.

Figure 2b is an equivalent observation for three microbubbles, all within 500 μm of each other at the time of focused ultrasound incidence, with full image sequence available as Supplemental Video S2 (online only). The inflation of the microbubble close to the capillary wall has been suppressed, likely because of its proximity to the wall, and perhaps shielding effects from the other two cavitation events. The jet from the centrally positioned upstream microbubble (to the left) is clearly directed toward the microbubble cavitation closer to the wall. Moreover, the jet direction from the on-axis microbubble has been steered approximately 5° anti-clockwise with respect to that from the relatively isolated microbubble of Figure 2a. Empirically, for focused ultrasound of PNP = 1.5 MPa (as the highest amplitude reported below), we estimate that a centrally located microbubble separated by >500 μm from the next closest microbubble may be considered relatively isolated in terms of jetting-direction.

Figure 3a(A-i) represents a S-V observation of the initial interaction between a relatively isolated on-axis microbubble (transducer *y*-position = 0 mm) and a burst of focused ultrasound at PNP = 1.5 MPa, with full image sequence at full FOV available as Supplementary Video S3 (online only). The image timings are further represented relative to hydrophone data for the pressure fluctuations of the first cycles of the focused ultrasound burst (Fig. 3b), measured before the capillary was in position. The quiescent microbubble initiating this activity is not well resolved at the start of Supplementary Video S3, possibly because it was of diameter below the spatial resolution of the S-V imaging, but more likely as it was too far removed from the imaging focal plane. A minor inflation to an *R*_max_ ≈ 5 μm (not represented in Fig. 3a) is in response to the first acoustic cycle from ∼0–5 μs (Fig. 3b), for which the pressure amplitudes are “ramping up” to the quoted value. The first significant inflation, to an *R*_max_ ≈ 45 μm, is captured at *t* = 7.9 μs (Figure 3a[A-i]) due to the rarefactional phase that peaks at *t* = 6.0 μs, with the inertia of the host medium imposing a delay to the cavitating microbubble response. The incoming compression phase peaking at 8.3 μs exerts a negative pressure gradient across the bubble (with respect to the positive x-direction [Fig. 1], with higher pressure to the left, lower to the right), with the asymmetry generating to a non-zero Kelvin impulse (Blake et al. 2015), and the formation of a jet (Ohl and Ikink, 2003, Rossellò et al., 2018) in the direction of the focused ultrasound propagation. The jet has sufficient momentum to neck and split from the main bubble at 10.6 μs, separating the gas phase for re-inflation under the action of the subsequent rarefaction. Figure 3b indicates that a positive pressure gradient (with lower pressure to the left, higher to the right) exists across the jetting bubble, during the re-inflation, which acts to flatten the jet tip from *t* ≈ 12.3 μs (apparent at *t* = 13.4 and 13.9 µs; Fig. 3a), initiating a rebound jet back through the main bubble, prominently visible at 14.6 μs. Rebound jets are also apparent in the T-V imaging Supplementary Videos S1 and S2, following the initial jets in the general direction of focused ultrasound propagation (Fig. 2).

**Fig. 4:**
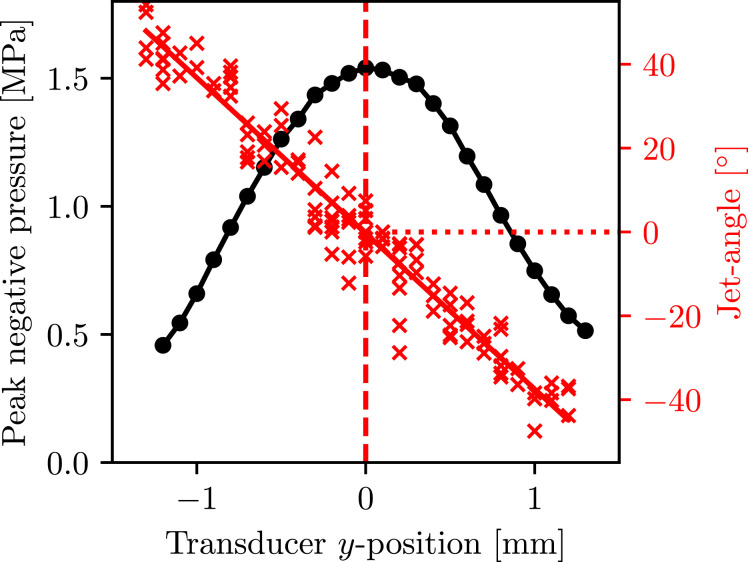
Plot of all data obtained for a burst of focused ultrasound with an on-axis peak negative pressure of 1.5 MPa, representing the relationship between initial microbubble-jetting angle (to the focused ultrasound propagation axis) and the lateral offset across the focus (transducer y-position). Peak negative pressure amplitude measurements for each offset location are also given.

Figure 3a(A-ii–vi), and Supplementary Videos S4–S8 (online only), respectively, represent microbubble response to an identical burst of focused ultrasound, but with the transducer elevated in the y-direction, such that the microbubble interacts with the acoustic focus at laterally offset positions from the propagation axis, from (ii) 0.4 mm to (vi) 1.2 mm. The PNP amplitudes measured for these, and all positions investigated, are given in Figure 4, which summarizes all directed-jetting data collected. Image row A at *t* = 7.9 μs represents the general trend for microbubbles inflating to reduced *R*_max_ values, for reduced PNPs moving away from the propagation axis. We note some variations for the exact timings of *R*_max_ at each location, mainly attributable to the inertia-imposed delay associated with each *R*_max_, and possibly also variations in the *R*_0_ values sampled from the SonoVue microbubble population (which are all apparent in Supplementary Videos S4–S8, but insufficiently focused for accurate *R*_0_ measurement), and focused ultrasound propagation times to each location. The main feature of Figure 3 is the downward turning of the initial jet, with increasing lateral offsets, as jetting (and rebound-jetting) follows the local pressure gradients, as described. The rebound jets of columns (v) and (vi), at transducer y-positions 1.0 and 1.2 mm, respectively, are not as prominent at *t* ≈ 14.6 μs as those for microbubble cavitation occurring closer to the propagation axis, (i)–(iv). This is because the inertia associated with the bubble oscillation is insufficient to sustain the re-inflation, for the positive pressure-gradient to act. Column (vi) further reveals that for the off-axis PNP = 0.5 MPa, the microbubble-cavitation re-inflations are quasi-spherical such that repeated jetting, at *t* = 8.9 and 14.3 μs, can occur. Image row M at 18.0 μs depicts the reducing degree of fragmentation experienced by the microbubble cavitation, across the reducing PNPs for increasing lateral offsets.

Figure 4 confirms that if the transducer is lowered with respect to the capillary (as opposed to elevated as for the data represented in Fig. 3a), the jet directions are turned upwards, symmetrically around the propagation axis.

**Fig. 5:**
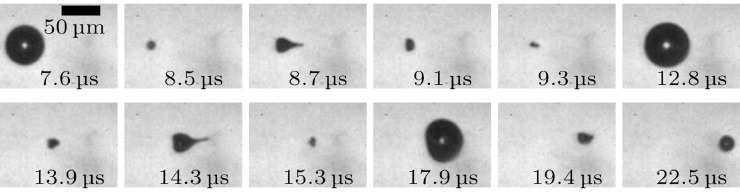
Representative images from a side view sequence of microbubble cavitation, aligned to the propagation axis, in response to a focused ultrasound burst with a peak negative pressure of 0.7 MPa.

Figure 5 represents the response of an on-axis microbubble to focused ultrasound, with the transducer at y-position = 0 mm and at a reduced PNP = 0.7 MPa, with full image sequence at full FOV available as Supplementary Video S9 (online only). The first *R*_max_ at *t* = 7.6 μs, occurs slightly earlier than the on-axis microbubble cavitation of Figure 3a(A-i), as the inertia-imposed delay associated with the smaller inflation, at lower PNP, is also reduced.

As for the off-axis microbubble cavitation of Figure 3a(A-vi), jetting is followed by quasi-spherical re-inflation, such that repeated jetting along with sudden intermittent translations in the direction of focused ultrasound propagation can occur. The frames at *t* = 9.3 and 15.3 μs show some indication that the microbubble cavitation is influenced by the positive pressure gradient, but that the rebound-jet effect is suppressed relative to Figure 3a(A-i), as the bubble is much smaller between the rarefactional and compressional phases of the propagating ultrasound.

## Discussion

Jetting from contrast agent microbubbles has received a significant level of attention because of the potential role that the dynamic may have in delivering drugs across biological barriers during microbubble cavitation-mediated therapy. Jetting through cell membranes has been reported (Prentice et al. 2005), as has jetting directed away from the inner, compliant, *ex vivo* vasculature wall, accompanied by strong tissue deformation (Chen et al. 2011).

In this study we investigated jetting from microbubbles flowing in a capillary, in response to a burst of focused ultrasound of frequency an order of magnitude below microbubble resonance and pressure amplitudes of interest for therapeutic applications. We found that initial microbubble response under such driving conditions is predisposed to jetting behaviour, generally consistent with considerations of the Kelvin impulse for liquid momentum (Blake et al. 2015). At PNP amplitudes $\tilde{>}$1 MPa, for which the initial inflation is sufficient that the inertia of the host medium sustains the inflation, the action of the pressure gradient between a compression and the successive rarefaction can also generate a prominent rebound jet. At lower PNP amplitudes, repeated jetting follows repeated quasi-spherical inflations, accompanied by sudden intermittent movements in the direction of focused ultrasound propagation. Moreover, directed jetting is observed, whereby initial jets from relatively isolated microbubbles “fan out” across the focus due to wavefront curvature, with an apparently linear dependence on lateral offset across the focus of ∼40°/mm, for the focused ultrasound field used in this work (Fig. 4).

Acoustically induced and directed jetting from bubbles generated *via* focusing a laser-pulse into a liquid (often termed *laser-induced cavities*) that is simultaneously hosting a high-amplitude acoustic field, with the bubble sufficiently distant from vessel walls to discount boundary effects, has previously been reported (Rossellò et al. 2018). A low-frequency acoustic source of 26 kHz was used for this work to influence the laser-cavity volumetric response (*R*_max_ values of several hundred microns), within viscous phosphoric acid solution. Bubble–acoustic pressure gradient interactions comparable to those reported here were observed; indeed, the evolution of the jetting bubble morphologies represented in Figure 3(a-i), is remarkably similar to those of Rossellò et al. 2018), albeit here an order of magnitude smaller and much more rapid. In another study (Gerold et al. 2012), a “jet fan” from single laser cavities induced across the focus of a higher-frequency (1.47 MHz) focused ultrasound field was observed, reminiscent of the directed microbubble-jetting above. It that case, however, the laser cavity volumetric oscillation was unaffected by the acoustic driving, with jets actuating due to cumulative radiation pressure across the bubble surface, over the duration of the collapse.

Finally, we note that transducers used for clinical development of transcranial blood–brain barrier disruption are large-aperture hemispherical arrays, generating approximately spherical focal regions (*e.g.,* Lipsman et al. 2018). For such a transducer geometry, radially directed microbubble jetting toward the focus for microbubbles in the near field, and away from the focus for microbubbles in the far field, may be expected. Future work will investigate the influence of compliant (tissue-representative) surfaces on jetting behaviour, particularly for repeated jetting as the microbubble cavitation approaches the boundary.

## Conclusions

Contrast agent microbubbles are predisposed to jetting behaviour during initial response to sub-megahertz focused ultrasound, such as that used for transcranial therapy of the brain. Jet characteristics depend on the pressure amplitude of, and pressure gradients within, the driving and proximity to other cavitating microbubbles.

## Appendix Supplementary materials

Supplementary Video S1 T-V imaging of the cavitation from a relatively isolated microbubble, in response to focused ultrasound of PNP = 1.5 MPa, a frame from which is represented in Figure 2a. Image, video 1

Supplementary Video S2 Top viewimaging of cavitation from three microbubbles, in response to focused ultrasound of peak negative pressure = 1.5 MPa, a frame from which is represented in Figure 2b. Image, video 2

Supplementary Video S3 Side view imaging of jetting activity from a single on-axis microbubble, at transducer y-position = 0 mm, frames from which are represented in Figure 3a(A-i). Image, video 3

Supplementary Video S4 Side view imaging of jetting activity from a single off-axis microbubble, at transducer y-position = 0.4 mm, frames from which are represented in Figure 3a(A-ii). Image, video 4

Supplementary Video S5 Side view imaging of jetting activity from a single off-axis microbubble, at transducer y-position = 0.6 mm, frames from which are represented in Figure 3a(A-iii). Image, video 5

Supplementary Video S6 Side view imaging of jetting activity from a single off-axis microbubble, at transducer y-position = 0.8 mm, frames from which are represented in Figure 3a(A-iv). Image, video 6

Supplementary Video S7 Side view imaging of jetting activity from a single off-axis microbubble, at transducer y-position = 1.0 mm, frames from which are represented in Figure 3a(A-v). Image, video 7

Supplementary Video S8 Side view imaging of jetting activity from a single off-axis microbubble, at transducer y-position = 1.2 mm, frames from which are represented in Figure 3a(A-vi). Image, video 8

Supplementary Video S9 Side view imaging of jetting activity from a single on-axis microbubble, at a peak negative pressure of 0.7 MPa, frames from which are represented in Figure 5. Image, video 9
